# Correction: Post-retrieval noradrenergic activation impairs subsequent memory depending on cortico-hippocampal reactivation

**DOI:** 10.7554/eLife.113156

**Published:** 2026-09-15

**Authors:** Hendrik Heinbockel, Gregor Leicht, Anthony D Wagner, Lars Schwabe

**Keywords:** Human

Heinbockel H, Leicht G, Wagner AD, Schwabe L. 2025. Post-retrieval noradrenergic activation impairs subsequent memory depending on cortico-hippocampal reactivation. *eLife* **13**:RP100525. doi: 10.7554/eLife.100525.

Published 29 January 2025

It came to our attention that the originally published figure 5 (panel C) contained a visualization error that may have been misleading. Specifically, while the x-axis was correctly arranged in ascending order from left to right, the corresponding GLMM plots were inadvertently displayed in the opposite orientation. This error occurred during the final preparation of the figure and was limited to the visualization; the underlying data, GLMM analyses, model estimates, statistical results, interpretations and conclusions are correct and remain unchanged. In the corrected figure, the GLMM plots have been reoriented to correspond to the ascending x-axis.

The corrected Figure 5 (updated panel C) is shown here:

**Figure 1:**
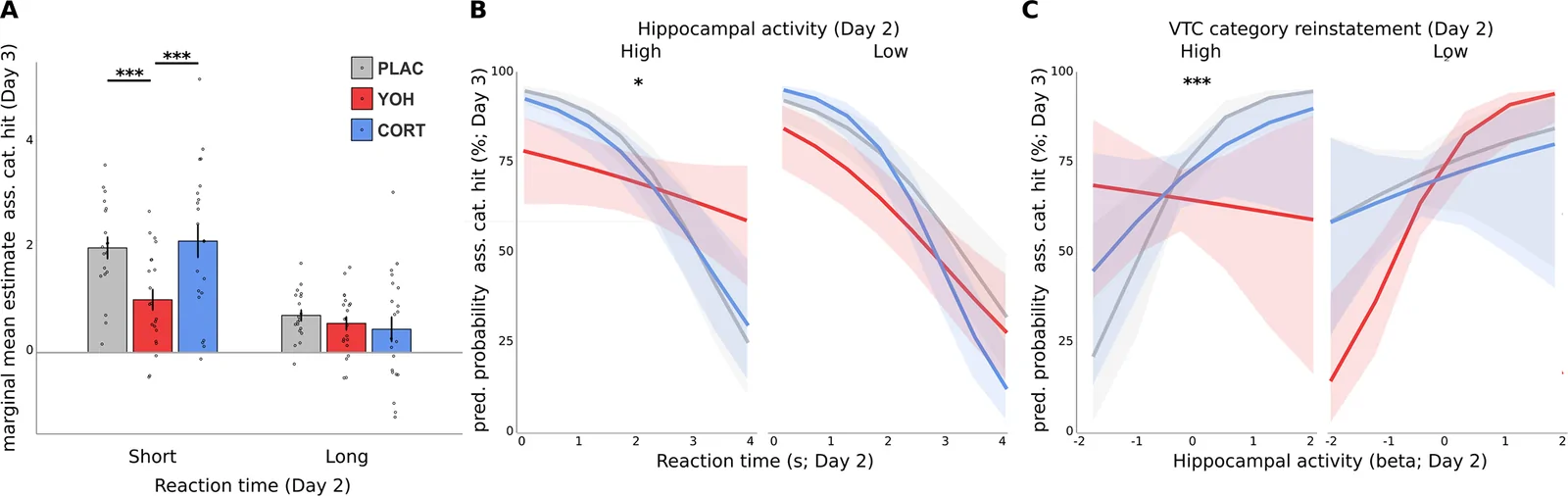


The originally published Figure 5 is shown for reference:

**Figure 2:**
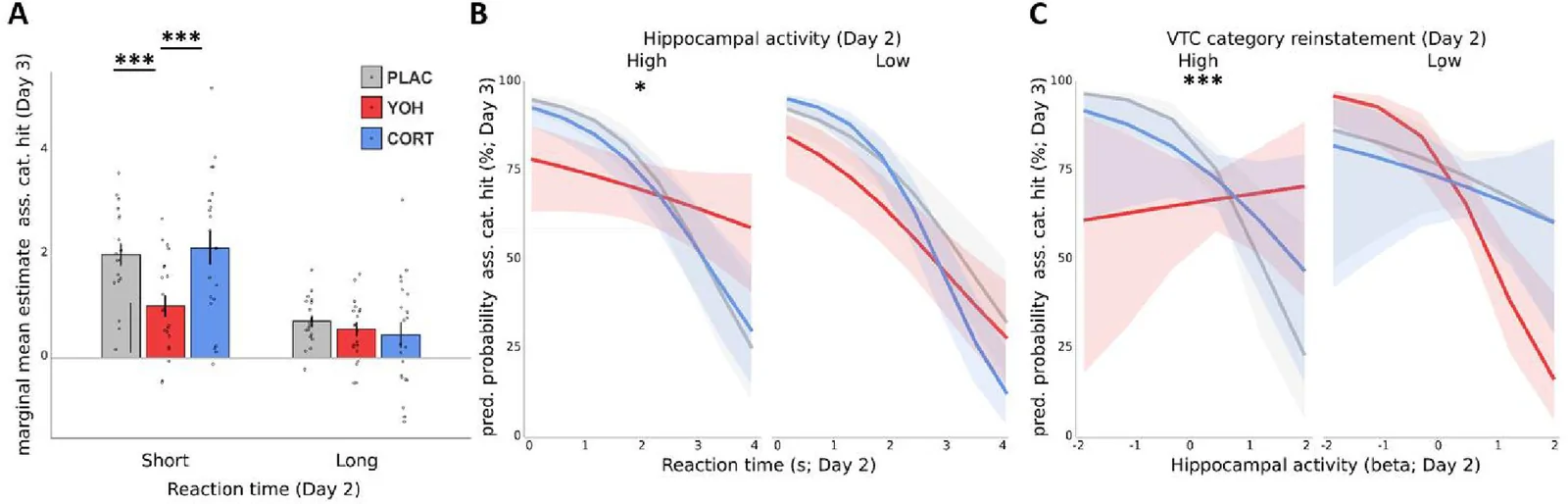


The article has been corrected accordingly.

